# Expanding the Armamentarium: Perspectives on Buccal Mucosal Grafts and Appendiceal Flaps in Ureteral Reconstructive Surgery

**DOI:** 10.3390/jcm14217681

**Published:** 2025-10-29

**Authors:** Dario Bello, Monica Van Shufflin, Matthias D. Hofer

**Affiliations:** 1 Department of Urology, University of Texas Health San Antonio, San Antonio, TX 78229, USA; 2 Urology San Antonio, San Antonio, TX 78229, USA

**Keywords:** augmented ureteral anastomosis, reconstructive surgical procedures, robotic surgical procedures, ureteral stricture

## Abstract

Management of complex and recurrent ureteral stricture disease remains one of the more challenging aspects of reconstructive urology. While standard techniques such as ureteroureterostomy, psoas hitch, Boari flap, and ileal ureter creation serve as the foundation of ureteral reconstruction, each technique has limitations, particularly when faced with recurrence, long strictures, and previously irradiated fields. Two alternative techniques—buccal mucosal graft (BMG) ureteroplasty and appendiceal onlay/interposition—have been previously described and are now being utilized more frequently in recent years. Furthermore, the advancement of robotic-assisted laparoscopic surgery has allowed for even more reconstructive capabilities. BMG ureteroplasty and appendiceal onlay/interposition can serve as valuable augmentations to the aforementioned surgical techniques. BMG has now long been established in urethral reconstruction and serves as a viable graft option for longer segment ureteral strictures, given its panvascular lamina propria and epithelium well-suited to a wet environment. Similarly, the appendix has other uses in urologic surgery, including the Mitrofanoff channel in pediatric surgery, and is chiefly used in right-sided ureteral stricture repair. Both of these techniques allow the surgeons to take on more complex ureteral stricture cases and avoid the morbidity of bowel harvest. In this perspective, we argue for a broader recognition and adoption of BMG and appendiceal onlay/interposition in ureteral reconstructive surgery. In this article, we highlight the rationale for use, technical considerations, outcomes, and clinical evidence suggesting their advantages over traditional approaches. By incorporating these techniques into practice, urologists can expand their ability to manage more complex ureteral stricture cases with improved outcomes.

## 1. Introduction

Ureteral stricture disease remains one of the more challenging aspects of reconstructive urology. There are many etiologies of ureteral strictures, including congenital anomalies, urolithiasis, prior endoscopic interventions, trauma, radiation-induced fibrosis, or iatrogenic injuries, and each can present unique challenges to management. Although most strictures are short and amenable to simple repairs or endoscopic management, repairs of longer or recurrent strictures are often more tenuous and require more refined methods or experienced hands. The decision of surgical approach requires a urologist to consider several factors, including the length/location of stricture, laterality, prior and medical/surgical history, with the goal to maximize the chances of ureteral patency, preserve renal function, and minimize morbidity to the patient.

Well-established reconstructive strategies for ureteral stricture disease include ureteroureterostomy, ureteroneocystostomy (with or without Boari flap/psoas hitch), ileal ureter substitution, and even renal autotransplantation (in severe cases). While each approach can be effective, each is limited by different anatomical criteria: primary ureteroureterostomy is often restricted to defects up to 3–4 cm in length, and ureteroneocystomy 4–5 cm in length for distal strictures (with psoas hitch and Boari flap providing an extra 6–10 cm and 12–15 cm of length, respectively). Ileal substitution is useful in very long stricture cases but can impose risks of metabolic derangements, bowel obstruction, and has relatively high long-term complication rates [1]. Renal autotransplantation has been studied more robustly in recent years with good success rates but remains technically challenging and is typically limited to high-volume centers with access to vascular services [2].

With these limitations in mind, the urologist must be able to adapt and rely on alternative management options when faced with complex or recurrent ureteral strictures. Owing to the advancement of robotic surgery in urology, both buccal mucosal graft (BMG) ureteroplasty and appendiceal onlay/interposition flaps have emerged as favorable options. Not only do these approaches avoid some of the morbidity of the aforementioned surgical options, but they also demonstrate high success rates in the literature. Improved visualization and dexterity enabled by robotic assistance allow urologists to be more accurate during these delicate reconstructive techniques. Furthermore, the use of fluorescence visualization (indocyanine green) provides an additional level of detail regarding tissue vascularization, which has proven to be key in the success of repair. This paper argues that BMG and appendiceal ureteroplasty are underutilized yet effective options that warrant broader consideration and incorporation into the reconstructive algorithm. We review the clinical evidence, technical aspects, advantages, and potential barriers, with the goal of advocating for their inclusion in routine urological practice.

## 2. Methods

A thorough literature review was conducted using PubMed (United States National Library of Medicine, Bethesda, MD, USA) to search for relevant publications between 2010 and 2025. The key terms used were “buccal mucosal graft”, “appendi-”, “ureteral reconstruction”, “ureteroplasty”, “ureteral stricture”, and “robotic reconstruction”. Individual publications were then selected based on relevance to the topic of this paper. Of the references that highlight treatment outcomes, it is important to consider the definition of treatment success, as it varies between studies. Success can be defined as either no obstruction on a nuclear medicine renal Lasix scan, improvement of hydronephrosis on a CT scan, symptom relief (flank pain, UTIs), serum creatinine improvement, no need for re-operation, or a combination of these.

## 3. Incidence and Etiologies of Ureteral Injuries/Strictures

The true incidence of ureteral strictures is difficult to quantify, given the heterogeneity of etiologies and reporting discrepancies. The incidence of iatrogenic ureteral injuries fluctuates between 0.3% and 1.5% in the literature [3]. Iatrogenic injuries and prior radiation treatment represent about 75% of all ureteral stricture cases [4]. Most iatrogenic cases occur during gynecologic surgery but may happen in colorectal and vascular surgeries in the pelvis as well [5]. While minimally invasive, endoscopic stone management also contributes to a rising incidence of strictures, particularly when thermal energy or ureteral dilation is used [6]. There are also several non-iatrogenic causes of ureteral strictures, including significant urolithiasis, retroperitoneal fibrosis, and congenital anomalies.

Important factors to consider in the management of ureteral stricture include the stricture length, location, and whether prior repair was attempted. Distal strictures are typically amenable to reimplantation into the bladder with adjunctive maneuvers (psoas hitch, Boari flap) as needed. Proximal and mid-ureteral strictures can be managed with direct ureteroureterostomy but pose a greater technical challenge, especially when longer than 3 cm [7]. Conventional solutions to these more complex cases would often require either intestinal substitution or even renal autotransplantation/nephrectomy in more severe cases. These methods do not go without long-term risks and morbidity, highlighting the need for reconstructive techniques that are both durable and limit morbidity.

## 4. Limitations of Conventional Reconstructive Options

Endoscopic techniques for ureteral strictures (including balloon dilation and endoureterotomy) are reasonable, minimally invasive options for treatment-naïve strictures less than 2 cm in length and for patients who are otherwise poor surgical candidates. They do, however, have much more variable success rates when compared to reconstructive techniques, making reconstruction the gold standard treatment [8].

**Figure 1:**
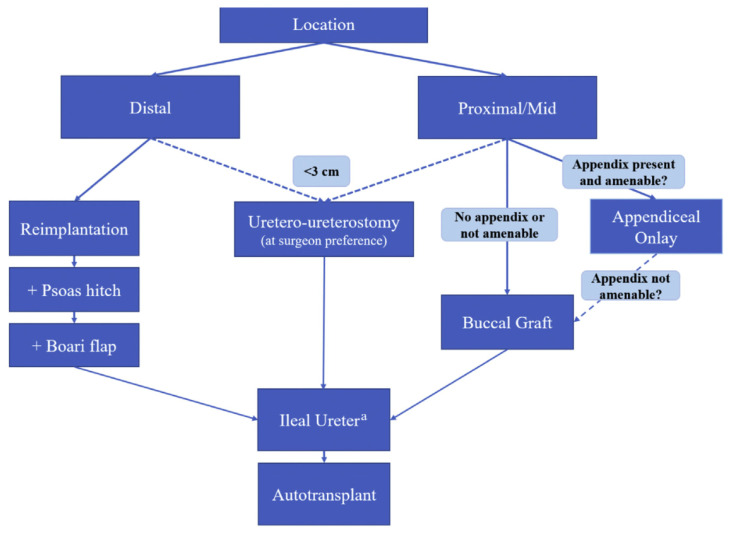
Ureteral reconstruction treatment algorithm as illustrated in Robotic Urologic Surgery [9].

Regarding treatment algorithms, the decision between endoscopic and reconstructive approaches is made through a shared decision-making approach. A provider must consider several factors, including the nature of the stricture disease and patient characteristics such as age, comorbidities, and willingness to undergo a major abdominal/pelvic surgery. In Robotic Urologic Surgery [9], a useful treatment algorithm is used when deciding on different reconstructive techniques for ureteral strictures (Figure 1).

Simple excision of a short stricture with direct end-to-end anastomosis is feasible in simple, short defects less than 2 cm in length. Attempting anastomosis after longer resections risks devascularization, tension on the anastomosis, and recurrence of stricture [10].

Psoas hitch and Boari flap maneuvers are reasonable approaches for longer, distal strictures requiring reimplantation, providing an extra 6–10 cm and 12–15 cm of length, respectively. However, they are limited to distal strictures and require the patient’s bladder to be of relatively normal capacity and compliance [11].

Ileal ureter substitution has long been described in the literature for long proximal strictures with durable patency. However, several complications, both short- and long-term, can arise, including small bowel obstruction, metabolic derangements, and mucus production. It remains a morbid procedure that requires longer hospitalization and may not be feasible in patients with prior abdominal/pelvic radiation or with prior bowel resections [12].

Renal autotransplantation preserves the renal unit and, while effective in specialized centers, is associated with complications such as vascular thrombosis, graft loss, and ureteral ischemia [2]. It is not widely available and may carry risks disproportionate to the disease burden in many patients.

Furthermore, any reconstructive technique is further complicated in previously irradiated or fibrotic fields. Instances of recurrence after previously attempted reconstruction can often leave the ureter and surrounding tissue ischemic and encased in fibrotic tissue. In such cases, alternative strategies are required for successful repair.

## 5. Buccal Mucosal Graft in Ureteral Surgery

Buccal mucosal grafts (BMG) have long been used within the realm of urologic reconstruction, particularly with regard to the urethra. This is due to its feasibility of harvest and compatibility with the urinary tract. It lacks hair growth, has viability within a moist environment that the urinary tract requires, and has excellent vascular supply via a pan-vascular lamina propria. Other tissue sites used that have been described in the literature, such as the use of bowel, present a higher risk of complications, comparatively. Therefore, BMG has become a favored reconstructive option within the urologic space.

Of course, BMG use does not go completely without risk, particularly within the harvest site (oral pain, scarring, infection, and sensory changes). Outside the harvest site, consideration includes urethral stricture recurrence secondary to graft ischemia, graft integration into the tissue site (depending on size and location), and limited use when attempting to reconstruct longer strictures. Despite the described risks, this surgical method continues to be considered a safe method with high success rates in urethral reconstruction. For this reason, its use has been considered for expansion, particularly in ureteral reconstruction. Alternative ureteral reconstructive options, as described within this paper, often come with increased invasiveness and, therefore, increased morbidity. This can be particularly true for those patients with more hostile abdomens that would make a major abdominal surgery, such as a bowel interposition, technically more difficult and with a lower success rate when compared to the use of BMG.

The use of BMG within the upper urinary tract has continued to emerge, and there have been a number of studies and series describing its use that show reasonable promise with high rates of success (>85%) and low rates of serious complications (<5%). Some notable studies that support this include the following:

– Heijkoop et al.’s systematic review (15 studies, 72 cases): pooled success ~92%, with complications in ~25% of patients but <5% being Clavien grade ≥ III [13].
– Chao et al. (10-year retrospective, 163 patients): robotic BMG ureteroplasty success rate of 92.0% [14].
– Lee et al. (54 patients, mean 27.5-month follow-up): 87% success, 5.6% major complications [15].

There are numerous other smaller studies, primarily within robotic use, that also support these findings.

Regarding its use in practice, BMG is most commonly used as an onlay graft but can also be used as a tubularized graft or hybrid with other adjuncts, such as in combination with omentum or peritoneum. Any of these can be completed via open, laparoscopic, or robotic approaches. As technology continues to advance, robotic approaches have been described more commonly in the literature and are thought to improve the precision of suture placement and tissue handling as well as improve accessibility in hostile abdomens [16,17].

Onlay grafts tend to be more commonly used as they allow some of the strictured ureter’s natural tissue to remain intact. This is performed by using the stretched graft tissue to lay over the partially excised stricture, working as a patch to enlarge the lumen. However, if end-to-end anastomosis is not possible, a tubularized graft can also be used. This, however, carries a higher risk than onlay graft devascularization due to a lack of native blood supply. This generally has been reserved for more complicated cases when other graft tissues are not feasible [18].

Ultimately, BMG ureteroplasty is a promising tool for ureteral reconstruction, in addition to its more commonplace use in urethral reconstruction. The literature review has shown a success rate of >85% at short-term follow-up intervals (1–3 years) with a <5% major complication rate [10,13,14,15,16,17,18]. Long-term data in the literature are still lacking; however, existing outcomes bode well for future studies (Table 1).

## 6. Appendiceal Onlay and Interposition in Ureteral Surgery

The appendix provides a well-vascularized, mucosa-lined conduit that is well-suited for ureteral reconstruction. Because it is already a tubular structure, it is very suitable as a flap for a tubular structure such as the ureter [20]. The use of the appendix in ureteral reconstruction dates back to 1912, when Melnikoff [21] first described its use. Since then, several studies and series have examined its use for ureteral reconstruction, particularly for more proximal strictures. Because of its location, the appendix is more suitable for right-sided stricture reconstruction, specifically.

**Figure 2:**
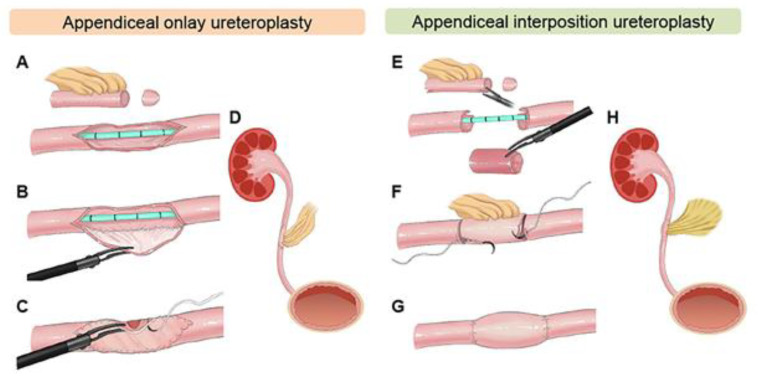
Appendiceal onlay (**A**–**D**) and appendiceal interposition surgical technique steps (**E**–**H**), as illustrated in Zhang et al. [22].

Two main applications exist for the appendix in ureteral reconstruction: one as an onlay flap, where the appendix is incised and used as a patch to augment the strictured ureter, and two as an interposition graft similar to ileal ureter substitution. The differing techniques are illustrated in Figure 2 [22]. The advantage here is that no bowel reconstruction is required, and minimal metabolic effects are associated with the patient. Furthermore, when the stricture is radiation-induced, the appendix may have been excluded from the radiation field, making it more likely to succeed in repair [23]. Appendiceal flaps also have some advantages when compared to buccal mucosal grafts, including inherent peristalsis that allows for the smooth passage of urine as well as a completely preserved blood supply Via the mesoappendix.

In regard to surgical technique, the appendix must first be identified and freed from its attachments to the cecum, taking care not to disturb its vascular supply. Then, as aforementioned, the tubular structure can be incised at its anti-mesenteric end to serve as a flap, or simply interposed between the two spatulated ends of the ureter (or bladder).

Several studies have demonstrated the success of appendiceal flaps for the use in ureteral reconstruction. Specifically in robotic-assisted laparoscopic cases, the success rates are favorable, ranging from 81 to 100%, with mean stricture length ranging from 2.5 to 6.5 cm in these studies (Table 2) [7,24,25,26,27,28]. In the largest of these studies, Cho et al. review a 14-patient cohort (aged 32 to 75 years) with a mean stricture length of 4.9 cm. 50% of these strictures were located proximally, with the etiology varying between iatrogenic, idiopathic, radiation-induced, urolithiasis, and prior endoscopic management (recurrence). In this series, ureteral stents were removed a median of 43.5 days after surgery. Treatment success was determined both radiographically (SFU grading of hydronephrosis) and clinically. 13 of the 14 (93%) patients demonstrated radiographic patency following stent removal, and no major complications were reported [7]. Wang et al. had the longest follow-up of the listed series, with a mean of 18 months, and satisfactory results. None of the patients required further surgical intervention and were considered successful, and no patients had major post-operative complications. 2 of the cases had residual mild hydronephrosis, although they were clinically asymptomatic [28].

Appendiceal interposition (end-to-end anastomosis) has been reported gradually in the literature over the years [27]. The use of appendiceal onlay flaps in ureteral reconstruction was first described in 2009 by Reggio et al. [29]. In a separate study, Duty et al. reported that the technique of onlay flap carries a lower risk of stricture recurrence compared to interposition [26]. Furthermore, robotic assistance in reconstructive surgery has enabled urologists with several advantages—magnified view, three-dimensional visualization, and improved articulation of instruments, which allow for more delicate and precise suturing.

There are inherent disadvantages to appendiceal flaps that must be considered. Firstly, the patient must not have had a prior appendectomy, and the appendix is most often reserved for right-sided ureteral strictures, especially in adults, due to its anatomical proximity and the limited mobility of the mesoappendix. The length of the appendix, whether used as an onlay flap or interposition, is also limited, so only ureteral strictures up to a certain length are amenable to repair.

Overall, the use of appendiceal onlay and interposition in ureteroplasty is a viable option for the repair of complex or recurrent ureteral strictures, particularly on the right side, due to its favorable tubular shape, vascular supply, and epithelial lining, while limiting morbidity related to bowel reconstruction.

## 7. Addressing Concerns, Barriers, and Limitations

The advantages of the robotic approach to reconstructive ureteral surgery are apparent, especially when augmented tissue flaps/grafts are used to aid in more complex or recurrent cases. Improved visualization and dexterity allow greater accuracy in suturing, as well as improved ergonomics, which are some of the reasons that allow for improved success rates. While the learning curve for robotics is typically shorter when compared to pure laparoscopy [30], we still must consider the typically increased complexity of cases when it comes to ureteral stricture disease. It is known that redo procedures are technically more challenging, largely due to extensive tissue reaction and fibrosis. Both BMG and appendiceal ureteroplasty require advanced reconstructive skills, particularly in regard to intracorporeal suturing and graft handling.

Patient selection is also key when considering surgical management options. Performing a thorough medical and surgical history will allow a provider to plan accordingly when taking on more complex ureteral stricture cases. In the case of using a graft/flap to augment repair, one must consider whether the patient has had prior radiation exposure, whether the appendix is still present, and any tobacco use or poor oral health (in the case of buccal mucosal grafts).

Specific to appendiceal flaps, as previously mentioned, one must consider the laterality of the ureteral stricture and length of the strictured segment, as the anatomy of the appendix and mesoappendix are limiting factors. For buccal mucosal grafts, one must consider the associated morbidity at the harvest site and ensure that the graft receives a good vascular supply at the repair site (which can be achieved with omental or perinephric fat flaps).

Lastly, surgeon training and experience must be considered. Ideally, the surgeon must have expertise in both robotics and reconstructive urology if they are to perform these more complex cases, hence why these patients are regularly referred to and treated at large tertiary centers or academic institutions, where fellowship-trained urologists are available.

One can make the case that proficiency in these techniques is attainable, even without fellowship training or residence at a large academic institution. As aforementioned, the learning curve for robotic surgery is favorable, so as these reconstructive techniques are used more frequently, there is an opportunity to expand the literature. The incorporation of these techniques into fellowship (and even residency) curricula and reconstructive societies will aid in broader adoption. Publications of standardized techniques, step-by-step videos, and multicenter registries would also further accelerate dissemination.

We must also consider the limitations within the existing literature. Of the aforementioned studies, the longest median follow-up time was 29 months. This is an important consideration, as ureteral stricture disease often recurs after several years. Studies with longer follow-up durations are needed to better encompass outcomes. While difficult to accomplish due to the heterogeneous nature of ureteral stricture disease, patient populations, and the relatively low volume of ureteral stricture cases performed, prospective studies comparing these newer augmented techniques to more traditional approaches would be ideal to compare outcomes.

## 8. Conclusions

Buccal mucosal graft and appendiceal onlay/interposition ureteroplasty represent innovative advances in the management of complex ureteral strictures. Both offer durable patency and low morbidity compared with more traditional techniques, as evidenced by their favorable success rates in the literature. Both have distinct advantages that make them favorable in cases of recurrence, longer stricture lengths, and even prior radiation exposure. While these techniques are typically reserved for salvage cases, they should be considered earlier in the treatment algorithm. Evidence in the literature is growing, but some gaps remain, particularly in the reporting of long-term follow-up. Given the sophistication of these techniques, future efforts should focus on standardizing technical aspects and expanding training opportunities. As evidence supporting their use grows, BMG and appendiceal ureteroplasty should be incorporated into guidelines and routine treatment algorithms, allowing urologists to offer patients safe and effective alternatives in complex ureteral reconstruction cases. As more data emerges, consideration should be given to include these new techniques within the guideline-driven ureteral stricture treatment algorithm.

## Figures and Tables

**Table 1:** Key studies of buccal mucosal graft (BMG) ureteroplasty.

Study (Year)	*N*	Median Stricture Length (cm)	Median Follow-Up Time (Months)	Success Rate (%)	Clavien–Dindo > 2 Complication Rate (%)	Notes
Zhao et al. (2018) [10]	19	4.0	26	90	0	
Heijkoop et al. (2021) [13]	72	1.5–11	2–85	92	5.0	Systematic Review
Lee et al. (2021) [15]	54	3.0	27.5	87	5.6	
Sahay et al. (2024) [17]	16	5.3	12	94	0	
Lee et al. (2024) [19]	10	2.5	10.3	80	0	
Chao et al. (2025) [14]	163	3.0	29	92	4.9	

**Table 2:** Outcomes of appendiceal onlay/interposition ureteroplasty.

Study (Year)	*N*	Mean Stricture Length (cm)	Mean Follow-Up Time (Months)	Success Rate (%)	Clavien–Dindo > 2 Complication Rate (%)
Duty et al. (2015) [26]	6	2.5	16	100	0
Jun et al. (2020) [24]	13	6.5	14.6	92	8
Burns et al. (2020) [25]	11	5	12	81	27
Wang et al. (2020) [27]	9	3.9	6.8	100	0
Wang et al. (2022) [28]	9	4.3	18	100	0
Cho et al. (2024) [7]	14	4.9	14.5	93	0

## Data Availability

No new data were created or analyzed in this study.

## Abbreviations

The following abbreviations are used in this manuscript:

BMG	Buccal Mucosal Graft

## References

[1] Nakada S.Y., Knoedler M.A., Best S.L., Dmochowski R., Kavoussi L., Peters C. (2025). Management of Upper Urinary Tract Obstruction. Campbell Walsh Wein Urology.

[2] Tran G., Ramaswamy K., Chi T., Meng M., Freise C., Stoller M.L. (2015). Laparoscopic nephrectomy with auto-transplantation: Safety, efficacy and long-term durability. J. Urol..

[3] Tyritzis S.I., Wiklund N.P. (2015). Ureteral strictures revisited…trying to see the light at the end of the tunnel: A comprehensive review. J. Endourol..

[4] Abboudi H., Ahmed K., Royle J., Khan M.S., Dasgupta P., N’Dow J. (2013). Ureteric injury: A challenging condition to diagnose and manage. Nat. Rev. Urol..

[5] Burks F.N., Santucci R.A. (2014). Management of iatrogenic ureteral injury. Ther. Adv. Urol..

[6] Delvecchio F.C., Preminger G.M. (2000). Management of ureteral strictures following ureteroscopy. J. Urol..

[7] Cho E.Y., Chaudry A.E., Puri D., Kim S., Viers B.R., Witthaus M., Buckley J.C. (2024). Outcomes of Robot-assisted Appendiceal Ureteroplasty From a Multi-institutional Experience. Urology.

[8] Lucas J.W., Ghiraldi E., Ellis J., Friedlander J.I. (2018). Endoscopic Management of Ureteral Strictures: An Update. Curr. Urol. Rep..

[9] Cheng N., Stifelman M., Wiklund P., Mottrie A., Gundeti M.S., Patel V. (2022). Reconstructive Surgery for Ureteral Strictures: Boari Flap, Psoas Hitch, Buccal Mucosa, and Other Techniques. Robotic Urologic Surgery.

[10] Zhao L.C., Weinberg A.C., Lee Z., Ferretti M.J., Koo H.P., Metro M.J., Eun D.D., Stifelman M.D. (2018). Robotic ureteral reconstruction using buccal mucosa grafts: A multi-institutional experience. Eur. Urol..

[11] Ahn M., Loughlin K.R. (2001). Psoas hitch ureteral reimplantation in adults--analysis of a modified technique and timing of repair. Urology.

[12] Launer B.M., Redger K.D., Koslov D.S., Sax-Bolder A.N., Higuchi T.T., Windsperger A.P., Flynn B.J. (2021). Long-term Follow Up of Ileal Ureteral Replacement for Complex Ureteral Strictures: Single Institution Study. Urology.

[13] Heijkoop B., Kahokehr A.A. (2021). Buccal mucosal ureteroplasty for the management of ureteric strictures: A systematic review of the literature. Int. J. Urol..

[14] Chao B.W., Raver M., Lin J.S., Zhao K., Lee M., Gelman S., Stifelman M., Zhao L.C., Eun D.D. (2025). Robotic Buccal Mucosa Graft Ureteroplasty: A Decade of Experience From a Multi-institutional Cohort. Urology.

[15] Lee Z., Lee M., Koster H., Lee R., Cheng N., Jun M., Slawin J., Zhao L.C., Stifelman M.D., Eun D.D. (2021). A Multi-Institutional Experience With Robotic Ureteroplasty With Buccal Mucosa Graft: An Updated Analysis of Intermediate-Term Outcomes. Urology.

[16] Nasef A.S., Tagrida I.A.E., Salman M.F., Elatreisy A., Khaled S.M. (2025). Buccal mucosal graft for onlay ureteroplasty in the management of proximal ureteral stricture. Arch. Ital. Urol. Androl..

[17] Sahay S.C., Kesarwani P., Sharma G., Tiwari A. (2024). Buccal mucosal graft ureteroplasty: The new normal in ureteric reconstructive surgery—Our initial experience with the laparoscopic and robotic approaches. J. Minim. Access Surg..

[18] Guliev B.G., Komyakov B., Avazkhanov Z., Shevnin M., Talyshinskii A. (2023). Laparoscopic ventral onlay ureteroplasty with buccal mucosa graft for complex proximal ureteral stricture. Int. Braz. J. Urol..

[19] Lee M., Nagoda E., Strauss D., Loecher M., Stifelman M., Zhao L. (2024). Role of buccal mucosa graft ureteroplasty in the surgical management of pyeloplasty failure. Asian. J. Urol..

[20] Doersch K.M., Hines L., Ajay D. (2024). Narrative review of flaps and grafts in robotic reconstructive urologic surgery. Ann. Laparosc. Endosc. Surg..

[21] Melnikoff A. (1912). Sur le replacement de l’uretere par anse isolee de l’in-testine grele. Rev. Clin. Urol..

[22] Zhang B., Chen J., Chen X., Liu L., He Y., Gan Y., Li B., Li Y., Long W., Han Z. (2025). Laparoscopic ureteroplasty for the treatment of long ureteral strictures with appendiceal interposition and appendiceal onlay flap: Technical description and initial experience. Res. Sq..

[23] Lee M., Lee Z., Metro M.J., Eun D.D. (2020). Robotic Ureteral Bypass Surgery with Appendiceal Graft for Management of Long-Segment Radiation-Induced Distal Ureteral Strictures: A Case Series. J. Endourol. Case Rep..

[24] Jun M.S., Stair S., Xu A., Lee Z., Asghar A.M., Strauss D., Stifelman M.D., Eun D., Zhao L.C. (2020). A Multi-Institutional Experience With Robotic Appendiceal Ureteroplasty. Urology.

[25] Burns Z.R., Sawyer K.N., Selph J.P. (2020). Appendiceal Interposition for Ureteral Stricture Disease: Technique and Surgical Outcomes. Urology.

[26] Duty B.D., Kreshover J.E., Richstone L., Kavoussi L.R. (2015). Review of appendiceal onlay flap in the management of complex ureteric strictures in six patients. BJU Int..

[27] Wang J., Xiong S., Fan S., Yang K., Huang B., Zhang D., Zhu H., Ji M., Chen J., Sun J. (2020). Appendiceal onlay flap ureteroplasty for the treatment of complex ureteral strictures: Initial experience of nine patients. J. Endourol..

[28] Wang J., Li Z., Fan S., Xiong S., Yuan C., Meng C., Zhang J., Zhang X., Zhang P., Ji M. (2022). Robotic ureteroplasty with appendiceal onlay flap: An update on the outcomes of 18-month follow-up. Transl. Androl. Urol..

[29] Reggio E., Richstone L., Okeke Z., Kavoussi L.R. (2009). Laparoscopic ureteroplasty using on-lay appendix graft. Urology.

[30] Yohannes P., Rotariu P., Pinto P., Smith A.D., Lee B.R. (2002). Comparison of robotic versus laparoscopic skills: Is there a difference in the learning curve?. Urology..

